# Water Extract of Mixed Mushroom Mycelia Grown on a Solid Barley Medium Is Protective against Experimental Focal Cerebral Ischemia

**DOI:** 10.3390/cimb43010030

**Published:** 2021-06-15

**Authors:** Ji Heun Jeong, Shin Hye Kim, Mi Na Park, Jong Yea Park, Hyun Young Park, Chan Eui Song, Ji Hyun Moon, Ah La Choi, Ki Duck Kim, Nam Seob Lee, Young Gil Jeong, Do Kyung Kim, Bong Ho Lee, Yung Choon Yoo, Seung Yun Han

**Affiliations:** 1 Department of Anatomy, College of Medicine, Konyang University, Daejeon 35365, Korea; jjh4110@konyang.ac.kr (J.H.J.); shinhye@konyang.ac.kr (S.H.K.); smcmjh@naver.com (J.H.M.); free136@naver.com (A.L.C.); rlejr19@nate.com (K.D.K.); nslee@konyang.ac.kr (N.S.L.); ygjeong@konyang.ac.kr (Y.G.J.); dokyung@konyang.ac.kr (D.K.K.); 2 Giunchan Co., Ltd., Pilot Plant, Cheonan 31035, Korea; guc2103@naver.com (M.N.P.); guc2203@naver.com (J.Y.P.); 3 Department of Occupational Therapy, College of Medical Science, Konyang University, Daejeon 35365, Korea; qkrgusdud1026@naver.com; 4 Department of Biomedical Laboratory Science, College of Medical Science, Konyang University, Daejeon 35365, Korea; sce0527@naver.com; 5 Department of Chemical Technology, Hanbat National University, Daejeon 34158, Korea; lbh011@hanbat.ac.kr; 6 Department of Microbiology, College of Medicine, Konyang University, Daejeon 35365, Korea; yc_yoo@konyang.ac.kr

**Keywords:** focal cerebral ischemia, *Phellinus linteus*, *Ganoderma lucidum*, *Inonotus obliquus*, solid-state fermentation, glutamate excitotoxicity

## Abstract

Although the individual consumption of medicinal mushrooms, including *Phellinus linteus* (PL), *Ganoderma lucidum* (GL), and *Inonotus obliquus* (IO), is known to be neuroprotective, the associated mechanisms underlying their therapeutic synergism on focal cerebral ischemia (fCI) have yet to be elucidated. This study aimed to demonstrate the neuroprotective effects of mixed mushroom mycelia (MMM) against experimental fCI. The water-fractions, ethanolic-fractions, and ethyl acetate-fractions of the MMM (PL, GL, and IO) grown in a barley medium using solid-state fermentation techniques were prepared and their protective effects against glutamate-induced excitotoxicity were compared in PC-12 cells. After the identification of the water extracts of MMM (wMMM) as the most suitable form, which possessed the lowest toxicity and highest efficacy, further analyses for evaluating the anti-apoptotic effects of wMMM, including Hoechst 33258-based nuclear staining, fluorescence-activated cell sorting, and reactive oxygen species (ROS) detection assays, were performed. Rats were subjected to a 90 min middle cerebral artery occlusion and reperfusion, after which a wMMM treatment resulted in significant dose-dependent improvements across a number of parameters. Furthermore, measurements of intracellular ROS and levels of antioxidant enzymes revealed a wMMM-mediated ROS attenuation and antioxidant enzyme upregulation. We suggest that wMMM is neuroprotective against fCI through its anti-apoptotic and anti-oxidative effects.

## 1. Introduction

Cerebral ischemia, mainly focal cerebral ischemia (fCI), is the second most common cause of death and the primary cause of long-term morbidity worldwide [1,2]. Cerebrovascular obstruction due to emboli or atherosclerotic plaques results in ischemic insults onto their parenchymal territories, where, consequently, reactive oxygen species (ROS) are accumulated and energy depletion-triggered necrotic cell death occurs. These initial events in the necrotic ischemic core eventually evolves into apoptotic cell death in the ischemic penumbra [3,4,5]. This mainly involves uncontrolled activation of post-synaptic neuronal N-methyl-D-aspartate receptors (NMDA-R) sensitized by glutamate, which is released from the necrotic presynaptic neuron [6]. The resulting over-activation of NMDA-R, which is calcium permeable, triggers intracellular calcium ([Ca^2+^]_i_) overload and causes the neuron to enter a vicious cycle towards apoptotic cell death [7,8]. This concept for the so-called “glutamate-induced excitotoxicity” is supported by the fact that fCI injury is reduced following supplementation with NMDA-R antagonists [9,10].

In addition to a series of events related to ROS accumulation-induced oxidative stress, neuro-inflammation, which is aggravated by adjacent microglia and astrocytes activated with neuronal apoptotic debris, is also known to be involved in fCI pathogenesis [11,12]. Upon the initiation of fCI, both neuronal apoptosis and neuro-inflammation led to a time-dependent propagation of neuronal damage from the ischemic core to the “penumbra” [5,13]. Therefore, it is imperative to develop effective drugs that therapeutically target ROS accumulation through their ROS scavenging activity or indirect activity, such as their upregulation of the expression levels of endogenous antioxidant enzymes and neuro-inflammation.

Among the candidates with anti-oxidative properties and anti-inflammatory properties, naturally originating bioactive compounds are currently attracting substantial attention. Among natural organisms, mushrooms, which are a subset of fungi, have not been as highly valued as plants. However, numerous studies over the past three decades have dealt with a broad spectrum of mushroom activities [14,15,16]. Mushrooms are widespread worldwide and are being increasingly appreciated not only for their unique taste and flavor but also as a source of bioactive compounds with health benefits, including antioxidant [17], antibacterial [18], antiviral [19], antifungal [20], antidiabetic [21], anti-inflammatory [22], and anticancer effects [23]. In addition, they reduce the incidence of cardiovascular diseases [24]. Their activities are known to be attributed to different groups of bioactive compounds such as polysaccharides, proteins, terpenoids, sterols, and polyphenols [25,26,27,28].

In the era of stroke research, medicinal mushrooms, which are defined as macroscopic fungi and defined as mostly higher Basidiomycetes, are revealed to possess significant neuroprotective effects in experimental settings [29,30]. Among medicinal mushrooms, *Phellinus linteus* (PL), *Ganoderma lucidum* (GL), and *Inonotus obliquus* (IO) are the most important species that have been widely consumed as oriental therapeutics [31]. All three species have been demonstrated to reduce the infarct volume and neurological deficits in fCI animal models when orally administered as a form of either fruit body or mycelium [32,33,34]. Based on these notions, we would expect a certain degree of therapeutic synergism when the PL, GL, and IO are administered in combination. However, due to their rareness in nature and the difficulties of cultivating them, their co-consumption has been considered difficult to implement, resulting in this type of research being quite scarce to date.

Due to modern solid-state fermentation (SSF) techniques, medicinal mushrooms are being rapidly and cost-effectively cultivated as a form of mycelia to meet market needs [35]. Fungal SSF on various types of grains used as a growth medium has been shown to exhibit a higher functional value than either grain substrates or mycelia themselves [36]. Most importantly, the production of mixed mushroom mycelia (MMM) is now theoretically feasible using SSF techniques.

**Figure 1:**
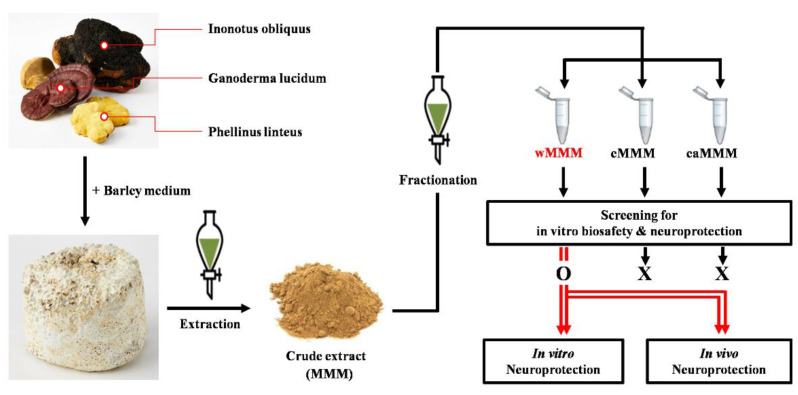
Time flow of this study. Barley flour, a growth medium, was inoculated with mycelia of *Phellinus linteus*, *Ganoderma lucidum*, and *Inonotus obliquus* yielding the mixed mushroom mycelia (MMM). The crude extract of MMM was further partitioned with the water-(wMMM), ethanolic-(eMMM), and ethyl acetate-extracts of MMM (eaMMM). Among the three extracts, the extract with the highest biosafety and neuroprotective efficacy were selected using an in vitro screening platform. After demonstrating wMMM as a feasible form, more detailed studies of in vitro neuroprotective effects and the possible in vivo neuroprotection of wMMM were carried out using PC-12 cells challenged with glutamate-induced excitotoxicity and a focal cerebral ischemia rat model, respectively.

This study aimed to demonstrate the neuroprotective effects of MMM (PL, GL, and IO) against experimental fCI. Barley flour was used as a growth medium for SSF in this study. After the acquisition of the water-(wMMM), ethanolic-(eMMM), and ethyl acetate-extracts of MMM (eaMMM), possible extracts with the highest biosafety and neuroprotection were screened using in vitro models of glutamate-induced excitotoxicity. Next, using in vitro and in vivo experimental fCI platforms, the neuroprotective effects and the possible therapeutic mechanisms of the selected extract form of MMM were studied (Figure 1).

## 2. Materials and Methods

### 2.1. Preparation of Extracts

MMM (PL, GL, and IO) and their different extract fractions (w-, e-, and eaMMM) were prepared and supplied by Giunchan Co., Ltd. (Cheonan, Korea). The mycelia of PL, GL, and IO were supplied by the Korean Forest Research Institute (Wanju, Korea) and inoculated in a potato dextrose agar (PDA) (Acumedia, Lansing, MI, USA) plate and incubated at 25 °C for 7 days. Following incubation, the mycelial disk was cut with a sterilized corn borer (8 mm) into 5–6 disks. The mycelium disks were then transferred to potato dextrose broth (Acumedia) and incubated with constant shaking at 25 °C for 7 days. Following incubation, the mycelia were homogenized in a stomacher (400 Mark Ⅱ, Seward Laboratory Systems Inc., Port St. Lucie, FL, USA) and then transferred to barley flour, which was immersed in distilled water for 1 h and sterilized. This was followed by incubation at 25 °C for 10 days. The resulting barley flour was inoculated with the 3 mycelia and the MMM was immersed in distilled water for 1 h and sterilized by autoclaving at 121 °C for 15 min. The MMM was then extracted by static maceration with ethanol (48 h, 1:2 *w*/*v*) and the residues were extracted with 70% ethanol (24 h, 1:1 *w*/*v*). The extracts were combined and the solvent was removed under reduced pressure at ≤45 °C to yield dried crude extract of MMM (extractive yield = 5.07%). Next, the crude extract was successively partitioned with the water-(wMMM, 3.93%), 70% ethanol-(eMMM, 1.06%), and ethyl acetate-extracts (eaMMM, 0.98%) according to the fractionation methods which can be found elsewhere [37].

### 2.2. Liquid Chromatography-Mass Spectrometry (LC-MS)

The chemical profiling of wMMM was carried out using a LC–MS instrument consisting of the Agilent 6200 series Time-of-Flight/6500 series (Agilent, Palo Alto, CA, USA) connected to the Agilent high performance liquid chromatography (HPLC) 1290 Infinity Binary Pump (Agilent) with an Electrospray ionization (ESI) interface. Zorbax Eclipse C18 column (5 µm, 150 mm × 2.1 mm) was used for chromatographic separation at a flow rate of 0.2 mL/min with two separate mobile phases. The mobile phase was a gradient system of high A in the first 3 min and high B in the next 15 min, followed by high A again (A: 0.1% formic acid in water and B: 0.1% formic acid in acetonitrile). The temperature of the column was maintained at 40 °C and the injection volume was 3 µL, with a total run time of 30 min. The spectra were obtained in ESI+ and ESI-modes and were acquired using a PDA detector. An Agilent Database Library was used to verify the compounds.

### 2.3. Cell Culture

PC-12 cells were purchased from the Korean Cell Line Bank (Seoul, Korea). PC-12 cells were cultured under a humidified incubator of 95% air and 5% CO_2_ at 37 °C with RPMI 1640 medium (Lonza, Walkersville, MD, USA) supplemented with heat-inactivated 10% fetal bovine serum (HyClone, Logan, UT, USA) and 1% penicillin/streptomycin mixture (Gibco, Waltham, MA, USA).

### 2.4. Cell Viability

PC-12 cells were seeded in a 96-well plate (1 × 10^4^ cells/well). In order to estimate the LD_50_ value of glutamate and neurotoxicity of the three types of MMM extracts, PC-12 cells were incubated with various concentrations of glutamate (0–30 mM) or the three types of MMM extracts (0–1000 μg/mL) diluted in a complete medium for 24 h and 16 h, respectively. After identifying the approximate LD_50_ value of glutamate and the common maximum safety concentration of all MMM extracts as 15 mM and 100 μg/mL, respectively, cells were co-treated with each extract and glutamate. The co-treatment was carried out by 16 h of pre-incubation with MMM extracts (10 or 100 μg/mL) and an additional 24 h of incubation with glutamate (15 mM). In order to assess any possible protection against glutamate-induced excitotoxicity, cell viability was measured using 3-(4,5-dimethylthiazol-2-yl)-2,5-diphenyl tetrazolium bromide (MTT) (Sigma, St. Louis, MO, USA) reduction assay according to the standard protocols found elsewhere [38]. The absorbance was measured at 570 nm with an ELx800 UV microplate reader (Bio Tek Instruments, Winooski, VT, USA). Data are expressed as the % of control values.

### 2.5. Hoechst 33258 Staining

PC-12 cells were seeded in the sterilized 12 Ø coverslip located in the 12-well plate bottom at a density of 1 × 10^5^ cells/well. After 24 h, the cells were co-treated with the wMMM and glutamate in accordance with the schedule described above (See Section 2.4). After the co-incubation, the cells were fixed with 4% paraformaldehyde (PFA) diluted in PBS for 1 h and stained with Hoechst 33258 (Sigma-Aldrich, St. Louis, MO, USA) diluted in H_2_O (1 mg/mL) at 23 °C for 15 min. After three washes in PBS, coverslips were mounted on slides and observed under an up-light microscope (DM4, Leica, Wetzlar, Germany). Under a fluorescence illumination setting (excitation/emission wavelength: 361/486 nm), the cells in a high-power field (HPF) showing bright and shrunken nuclei, a hallmark of ongoing apoptosis, and were counted and averaged. Observations of over 10 HPFs that were randomly selected were executed for this experiment. The experiments and the data acquisitions were carried out in triplicate.

### 2.6. Flow Cytometry

In order to quantify PC-12 cell apoptosis in response to the different treatments, Muse^TM^ Cell Analyzer (Merck-Millipore, Burlington, MA, USA), an automated fluorescence-activated cell sorting (FACS) device, and the corresponding Annexin V-FITC Apoptosis Detection Kit (Merck-Millipore, Burlington, MA, USA) were used. The Annexin V-FITC Apoptosis Detection Assay Kit utilizes a fluorescein isothiocyanate (FITC) conjugated to Annexin V as an apoptotic cell marker and 7-AAD as a dead cell marker. To this end, cells were co-treated with the wMMM and glutamate in accordance with the schedule described above (See Section 2.4). The experiment was carried out according to the protocols provided by the manufacturer. The data and scatter plots were automatically obtained from software embedded in the device. The experiments and the data acquisitions were carried out in triplicate.

### 2.7. 2′, 7′-Dichlorodihydrofluorescin Diacetate (DCF-DA) Assay

The intracellular ROS level was monitored using a DCF-DA (Sigma-Aldrich, St. Louis, MO, USA). In brief, PC-12 cells seeded in a 12-well plate (1 × 10^5^ cells/well) were pretreated with or without wMMM extract (10 or 100 μg/mL) for 16 h, treated with 100 μM DCF-DA diluted in complete medium for 30 min, washed with PBS, and challenged with or without 15 mM glutamate diluted in complete medium for 10 min. At the end of the incubation, the resulting fluorescence was detected using a fluorescence live-cell movie analyzer (JuLI-FL^®^, Pleasanton, CA, USA) at excitation/emission wavelengths of 395/509 nm. The experiments and the data acquisitions were carried out in triplicate.

### 2.8. Animals

Male Sprague-Dawley rats (220–250 g, 8 weeks) were purchased by Samtako (Osan, Korea). Rats were maintained at a controlled temperature (22–23 °C) and humidity (40–60%) according to 12:12 h light/dark cycles with access to food and water *ad libitum*. Experiments were executed in accordance with the Guide for the Care and Use of Laboratory Animals (*National Institutes of Health publication, 8th Edition*, 2011) [39]. Animal experiments in this study were approved by the Institutional Animal Care and Use Committee of Konyang University (Daejeon, Korea; approval code, P-21-11-A-01; approval date, 24 March 2021).

### 2.9. In Vivo Experimental Plan

Rats were stabilized for 7 days and randomly divided into four groups, as follows (n = 12 per group): The control (Cont) group without wMMM treatment and middle cerebral artery occlusion and reperfusion (MCAO/R); the operated (OP) group with vehicle (water) and MCAO/R; the wMMM-L group with low dose (30 mg/kg) of wMMM and MCAO/R; the wMMM-H group with high dose (90 mg/kg) of wMMM and MCAO/R. The wMMM that was dissolved in a vehicle to obtain a final volume of 1 mL or the equivalent volume of vehicle was administered by an intragastric route for 4 times prior to MCAO/R in a daily regimen. For the randomly selected rats (n = 2 per group) assigned to an in vivo ROS detection assay, dihydroethidium (DHE) was injected into the jugular vein just prior to MCA occlusion during the MCAO/R and sacrificed 2 h after the operation for brain sampling (See Section 2.16). The other randomly selected rats (n = 4 per group) assigned to a western blot were sacrificed at 2 h after the operation to obtain brain homogenates (See Section 2.17). The remaining rats (n = 6 per group) underwent four different behavioral tests (See Section 2.11) at 24 h after the MCAO/R and were then sacrificed for infarct volume measurement (See Section 2.12) and subsequent histologic studies (See Section 2.13, Section 2.14 and Section 2.15). The time flow of the in vivo experimental plan in this study is depicted in Figure 4A.

### 2.10. Middle Cerebral Artery Occlusion and Reperfusion (MCAO/R)

The fCI was induced by MCAO/R according to the operational procedures previously described [40]. Briefly, rats were anesthetized with 1.5% isoflurane and a gas mixture of oxygen and nitrous oxide (28.5:70) under the control of body temperature (37 ± 0.5 °C) using a heating pad connected with a rectal probe (JD-DT-08-06, Jeong-do B&P, Seoul, Korea). After a midline incision in the neck, the left common carotid artery (CCA) and the external carotid artery were ligated with 4-0 black silk. Then, the internal carotid artery (ICA) was transiently occluded with an aneurismal clip. A small hole in the CCA was made by micro-scissors and 4-0 nylon monofilaments with a round silicon-coated tip (tip diameter: 0.35–0.37 mm) (403534PK10, Doccol, Sharon, MA, USA) were inserted. After eliminating the aneurismal clip, the silicon-coated filament was advanced into the ICA until the relative cerebral blood flow (rCBF) was dropped below 20% of the baseline under the guidance of a Doppler flowmeter (Periflux 5000, Perimed Inc., Järfälla, Sweden). After subjecting the rats to a 90 min ischemic period, the filament was withdrawn and reperfusion began. The surgical wounds were closed and the rats were returned to their home cage.

### 2.11. Behavioral Tests

The neurological deficit scoring (NDS), Rotarod, a grip strength test, and inverted screen tests were executed at 24 h after the MCAO/R to compare the fCI-induced sensorimotor deficits of different groups according to the previously described protocols [41,42,43]. First, for NDS, the scoring was carried out based on a “4-point scale” as follows: 0, no neurologic deficit; 1, failure to spread out affected forepaw; 2, unidirectional circling; 3, falling to one side; and 4, no voluntary movement. Second, for the Rotarod, rats were placed on the rotating drum (15 rpm, a constant velocity mode) over 300 s and their latency to fall were recorded. Third, for the grip strength test, the rats were permitted to grab the bar connected to the grip strength meter with both forepaws and the maximum force when the grip was released upon pulling them back was measured. Lastly, the inverted screen test was used according to the standard protocol with a slight modification in the form of an inclination angle changed from 180° to 90°. In this test, the rats were allowed to grip the metal grid and the grid was gradually inclined to the desired angle, 90°. The latencies to fall onto soft bedding were then recorded up to 180 s. All recordings in the aforementioned tests were carried out in triplicates by blind observers and the data were averaged across groups.

### 2.12. Infarct Volume Measurement

After all behavioral tests, the rats were decapitated and their whole brains were quickly isolated. The brains were cut into seven coronal slices (2 mm in thickness) along their cranio-caudal axis. The slices were stained in 1% triphenyltetrazolium chloride (TTC) solution, diluted in PBS at 37 °C for 30 min, and fixed with 4% PFA for 2 h. The continuously sectioned brain slices were sequentially aligned and photographed and the area of infarction (white area) was analyzed using image J (Version 1.53 g, National Institutes of Health, Bethesda, MD, USA). The infarct area was calculated according to the following formula: the ipsilateral hemispheric infarct area/the contralateral hemispheric area×100. The resulting areas were summed up to yield the ipsilateral infarct volumes of the rats and averaged across groups.

### 2.13. Cresyl-Violet (C-V) Staining

The fourth TTC-stained slice in a cranio-caudal axis was used for histologic studies. To this end, excessive fixatives remaining in the tissue were quickly removed and the tissues were dehydrated using a graded ethanol series that were embedded in paraffin and sliced into 5 μm sections using a microtome (RM2255, Leica, Wetzlar, Germany). After deparaffinization using xylene, the number of viable neurons in the penumbral region was examined by cresyl-violet (C-V) stain according to the standard protocols found elsewhere [44,45]. The three randomly chosen HPFs (400×) per C-V-stained tissue slides were photographed by DM4 light microscopy (Leica, Wetzlar, Germany) and the number of viable neurons was averaged across groups. A neuron with a visible nucleolus was considered a viable neuron.

### 2.14. Terminal Deoxynucleotidyl Transferase dUTP Nick-End Labeling (TUNEL) Assay

Cells undergoing apoptosis were quantitatively assessed using a TUNEL assay kit (DeadEnd^TM^, Promega, Madison, WI, USA). Deparaffinized tissue sections prepared as described above were sequentially treated with the reagents contained in the kit according to the protocols provided by the manufacturer. At the end of this procedure, coverslips were mounted on a sliding glass and visualized by DM4 up-light microscopy (Leica, Wetzlar, Germany). Under a fluorescence illumination setting (excitation/emission wavelength: 395/509 nm), three HFPs (400×) per rat were randomly chosen and the TUNEL-positive cells were averaged across groups.

### 2.15. Immunohistochemistry

Deparaffinized tissue sections prepared as described above were reacted with a tissue retrieval solution for 5 min at 120 °C, after which their endogenous peroxidase activity was blocked using 1% hydrogen peroxide for 1 h at 22 °C. Following three PBS washes, slides were incubated with rabbit Cleaved caspase-3 antibody (Cell signaling, Denver, MA, USA) diluted in PBS (1:200) in a humid chamber for 24 h at 4 °C. After PBS washes, the slides were incubated with biotinylated anti-rabbit IgG (Vector, Burlingame, CA, USA) diluted in PBS (1:250) for 2 h at 22 °C. Information regarding product number of the antibodies used in this study are presented in supplementary material (Table S1). After washes in PBS, the slides were reacted with a VECTASTAIN-Elite avidin-biotin complex Kit (Vector, Burlingame, CA, USA) for 30 min at 22 °C. In order to visualize the immunoreactivities, the slides were reacted with the chromogen 3,3’-Diaminobenzidine (DAB) (Vector, Burlingame, CA, USA) for 30 min at 22 °C. The three HFPs (400×) per rat were randomly chosen and visualized by DM4 up-light microscopy (Leica, Wetzlar, Germany). The number of the cleaved caspase-3-immunoreactive cells (dark-brown colored) was averaged across groups.

### 2.16. Dihydroethidium (DHE) Assay

The extent of brain ROS was quantified using in vivo perfusion of DHE (Invitrogen, Carlsbad, CA, USA). In brief, 10 mg/kg DHE diluted in 50 μL of PBS was injected into rats via their jugular vein just prior to MCA occlusion during the MCAO/R. At 2 h after reperfusion, the brain was quickly removed and immersed in a 30% sucrose solution for cryopreservation, embedded in the optimal cutting temperature (OCT) compound, and frozen (−80 °C). The brains were cut at a thickness of 15 μm using a cryostat microtome (CM1850, Leica, Wetzlar, Germany) set to −21 °C. Three randomly selected sections from each rat were counter-stained with Hoechst 33258 (Sigma-Aldrich, St. Louis, MO, USA) for 15 min at 22 °C and cover slipped. In three randomly chosen HFPs (400×) per rat, DHE-fluoresce cells (red) were photographed by laser scanning confocal microscopy (LSM-700; Carl Zeiss, Oberkochen, Germany). The fluorescence intensity was measured by Image J (Version 1.53g) and averaged across groups.

### 2.17. Western Blot Analysis

Tissue samples (n = 4 per group) were obtained from the ipsilateral striatum and cerebral hemisphere at 2 h after MCAO/R. The total protein of the tissue was obtained after these tissues were homogenized in a lysis buffer containing protease inhibitors. Thereafter, the protein concentration of each sample was estimated using the Bicinchoninic acid (BCA) assay kit (Thermo-Fisher, Waltham, MA, USA). Proteins (30 μg) were separated by electrophoresis on 10% sodium dodecyl sulfate-polyacrylamide gels (SDS-PAGE) and transferred onto a Polyvinylidene fluoride (PVDF) membrane (Merck-Millipore, Burlington, MA, USA). After blocking for 1 h at 22 °C with 5% skim milk diluted in Tris-buffered saline with Tween 20 (TBS/T), membranes were incubated for 24 h at 4 °C with the primary antibodies: anti- superoxide dismutase (SOD; Sigma-Aldrich, St. Louis, MO, USA), anti- heme oxugenase-1 (HO-1; Abcam, Cambridge, UK), anti-catalase (CAT; Sigma-Aldrich, St. Louis, MO, USA), and anti-β-actin (Santa Cruz, Dallas, TX, USA). All primary antibodies were diluted in a blocking solution at a ratio of 1:1000. After washing with TBS/T, membranes were incubated for 2 h at 23 °C with horseradish peroxidase (HRP)-conjugated Anti-IgGs (Invitrogen, Carlsbad, CA, USA) as the secondary antibodies. All secondary antibodies were diluted in a blocking solution at a ratio of 1:2000. Information regarding product number of the antibodies used in this study are presented in supplementary material (Table S1). Antigens were detected using the standard chemiluminescence method (ECL; Merck-Millipore, Burlington, MA, USA). Quantification of the protein band was carried out using Image J software (Version 1.53g). Protein levels were normalized to those of β-actin and normalized to those of the Cont group.

### 2.18. Statistical Analysis

Data were analyzed and plotted using GraphPad Prism (Graph-Pad Software, San Diego, CA, USA). Statistical analysis was performed using a one-way ANOVA, followed by a Tukey post hoc test for pairwise comparisons. All data were presented as the mean ± standard deviation (SD). Values of *p* < 0.05 were considered statistically significant.

## 3. Results

### 3.1. Water Extracts of Mixed Mushroom Mycelia (wMMM) Protects PC-12 Cells against Glutamate-Induced Excitotoxicity by Having the Highest Biosafety and Efficacy among All MMM Fractions

**Figure 2:**
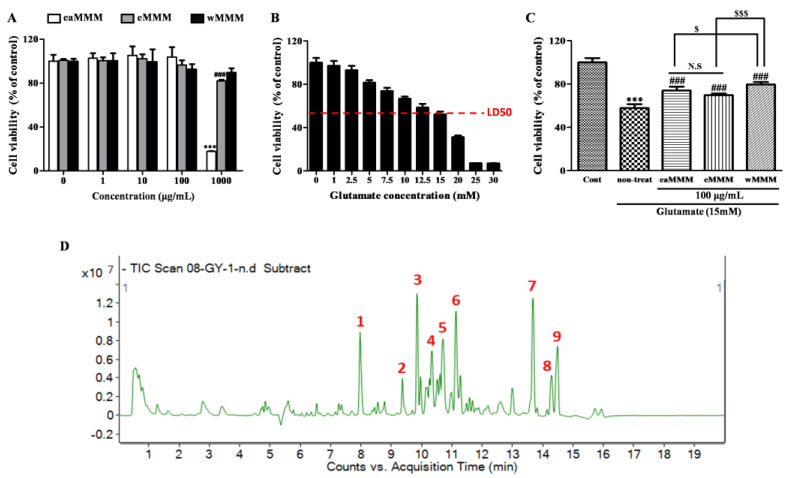
Selection of water extracts of mixed mushroom mycelia (wMMM) as a feasible form of MMM with the highest biosafety and neuroprotective efficacy and chemical profiling. In order to evaluate the biosafety of w-, ethanolic-(e-), and ethyl acetate-extract-(ea-) MMM, (**A**) the cell viabilities of PC-12 cells following incubation with the varying concentrations (0-1000 μg/mL per each) for 16 h were analyzed by a 3-(4,5-dimethylthiazol-2-yl)-2,5-diphenyl tetrazolium bromide (MTT) assay (*** *p* or ^###^ *p* < 0.001 vs. the corresponding non-treated controls). In order to identify the LD_50_ of glutamate, (**B**) the cell viability following 24 h of incubation with varying concentrations of glutamate (0–30 mM) were assayed (cell counts value reduced to 50% was depicted with red line). In order to evaluate neuroprotective efficacy, (**C**) cell viability was analyzed in the PC-12 cells co-treated with three fractions of MMM and glutamate (*** *p* < 0.001 vs. Cont; ^###^ *p* < 0.001 vs. glutamate non-treated; ^$$$^ *p* < 0.001 vs. eMMM; ^$^ *p* < 0.05 vs. eaMMM; N.S; not significant). The co-treatment was accomplished by 16 h of pre-incubation with MMM extracts (100 μg/mL) and an additional 24 h of incubation with glutamate (15 mM). In all graphs (**A**–**C**), data are represented as the mean ± standard deviation (n = 8 per condition). Significance was determined by one-way ANOVA followed by a Tukey post hoc test for pairwise comparisons. (**D**) Chemical spectra of wMMM were obtained by liquid chromatography-mass spectrometry.

In order to select the most feasible form of the MMM extract for further in vitro and in vivo experiments, the crude extract of MMM was initially fractionated into wMMM, eMMM, and eaMMM according to the schematic diagram shown in Figure 1. The different extracts were subjected to PC-12 cell-based screening platforms to test their neuroprotective efficacy. Prior to these, the cytotoxicities of each extract at 16 h of the incubation period were assessed (Figure 2A). While the eaMMM and eMMM showed cytotoxicity, the wMMM had no effect on PC-12 cell viability at a concentration of 1000 μg/mL. These data indicated that, among the three MMM extracts, wMMM was the formulation with the highest biosafety. The following cell viability test revealed that 15 mM was the approximate median lethal dose (LD_50_) of glutamate at 24 h of incubation in our experimental setting (Figure 2B). As all extracts were revealed to be nontoxic below at 100 µg/mL, this concentration was used to compare the extracts’ protective efficacies against glutamate (15 mM; LD_50_)-induced excitotoxicity in PC-12 cells (Figure 2C). Results revealed that all MMM extracts exhibited a neuroprotective effect against glutamate-induced excitotoxicity (^###^ *p* < 0.001). Notably, at this concentration (100 µg/mL), wMMM showed the highest efficacy (^$$$^ *p* < 0.001 vs. eMMM and ^$^ *p* < 0.05 vs. eaMMM). In order to determine the compounds that are prevalent in the wMMM, we performed chemical profiling using LC-MS. The total ion current chromatogram of the wMMM revealed nine main peaks (Figure 2D). The following verification process comparing their retention time with an Agilent Database Library revealed that the corresponding constituents of wMMM were suspected to be Sanleng acid, 9-Ene-methyl palmitate, Dibutyl sebacate, Matairesinoside, 19-Glucosyl-14-deoxyandrographolide, Vernolic acid, 6-Gingerol, n-Henicosanal, and Kingianoside B (Table 1).

### 3.2. Water Extracts of Mixed Mushroom Mycelia (wMMM) Protects PC-12 Cells against Glutamate-Induced Excitotoxicity by Reducing Apoptosis and Oxidative Stress

**Figure 3:**
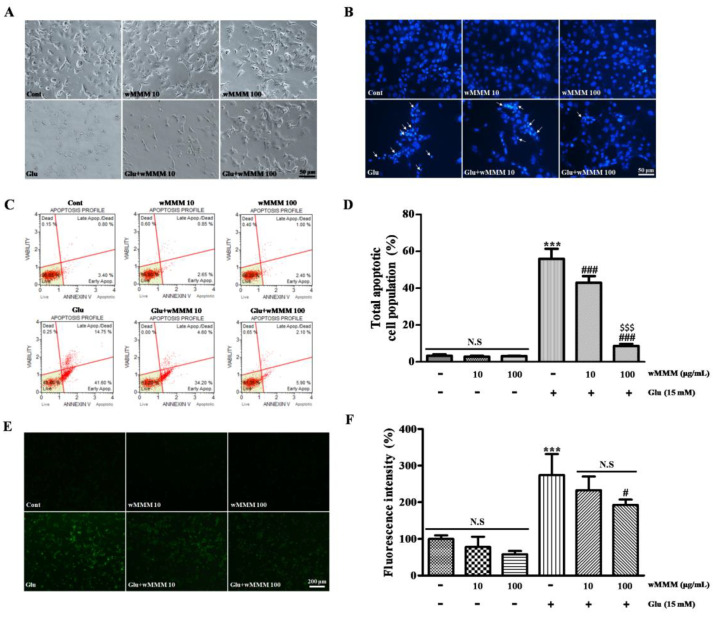
Anti-apoptotic and anti-oxidative effects of water extracts of mixed mushroom mycelia (wMMM) on PC-12 cells challenged with glutamate-induced excitotoxicity. Representative cell morphologies (**A**) and Hoechst 33258-stained nuclei (**B**) captured by inverted microscopy. Shrunken and illuminating nuclei depicted with white arrows. Representative dot plot (**C**) obtained by Annexin V-FITC/7-AAD-based flow cytometry and bar graphs (**D**) quantifying the ratio of apoptotic cells (early and late apoptotic/dead cells) per total cells are shown. Representative 2′, 7′-dichlorodihydrofluorescin diacetate (DCF-DA)-stained fluorescence images (**E**) and the bar graphs quantifying the fluorescence intensities (**F**). For (**A**–**D**), the co-treatment was accomplished by 16 h of pre-incubation with MMM extracts (10 or 100 μg/mL) and an additional 24 h of incubation with glutamate (15 mM). For (**E**,**F**), the co-treatment was carried out by 16 h of pre-incubation with MMM extracts (10 or 100 μg/mL) and an additional 10 min of incubation with glutamate (15 mM). In both graphs (**D**,**F**), data are represented as the mean ± standard deviation (*** *p* < 0.001 vs. cells without wMMM and glutamate; ^#^ *p* < 0.05 and ^###^ *p* < 0.001 vs. cells treated with glutamate only; ^$$$^ *p* < 0.001 vs. cells co-treated with 10 μg/mL wMMM and glutamate; N.S, not significant). In all graphs, significance was determined by one-way ANOVA followed by a Tukey post hoc test for pairwise comparisons. The experiments and data acquisitions were carried out in triplicates.

After identifying wMMM as the most feasible to utilize in further studies, we tested whether wMMM could attenuate the apoptosis of PC-12 cells challenged with glutamate-induced excitotoxicity. Although cellular (Figure 3A) and nuclear morphology (Figure 3B) remained unaltered by incubation with both 10 μg/mL or 100 μg/mL of wMMM, apoptotic features of general appearance (scarce cell number and retraction of the cellular process) and nuclear morphology (shrunken and illuminating nuclei upon Hoechst 33258 staining) were prominent in the cells treated with glutamate (15 mM). However, the cells co-treated with 10 μg/mL or 100 μg/mL of wMMM were relatively free from the structural deteriorations induced by a glutamate insult. In order to quantify the modulatory effect of wMMM on apoptosis, we performed a FACS-based apoptosis assay (Figure 3C). Results demonstrated that glutamate markedly increased the cellular fractions’ ongoing apoptosis compared with the untreated controls (55.93 ± 4.40 vs. 3.26 ± 0.74, *** *p* < 0.001, Figure 3D). However, co-treatment with 10 μg/mL or 100 μg/mL wMMM could reverse this (42.95 ± 2.94 and 8.59 ± 0.94, respectively; ^###^ *p* < 0.001 vs. glutamate) in a dose-dependent manner (^$$$^ *p* < 0.001). Given that oxidative stress is known to be the key upstream factor linked to excitotoxicity-induced neuronal apoptosis and a vast majority of bioactive ingredients originated from mushroom mycelia are reported to exert anti-oxidative property in the previous literature [46,47], we next assessed whether the wMMM could affect the glutamate-induced accumulation of intracellular ROS using a fluorescent probe DCF-DA assay (Figure 3E). In line with the FACS results, glutamate markedly increased the intracellular ROS levels (274.09 ± 49.87% of the untreated control, *** *p* < 0.001; Figure 3F). Although 10 μg/mL wMMM failed, 100 μg/mL wMMM could significantly inhibit the increase in intracellular ROS (192.65 ± 12.66%, ^#^ *p* < 0.01 vs. glutamate).

### 3.3. Water Extracts of Mixed Mushroom Mycelia (wMMM) Diminishes Focal Cerebral Ischemia (fCI)-Induced Infarct Volume and Behavioral Deficits

**Figure 4:**
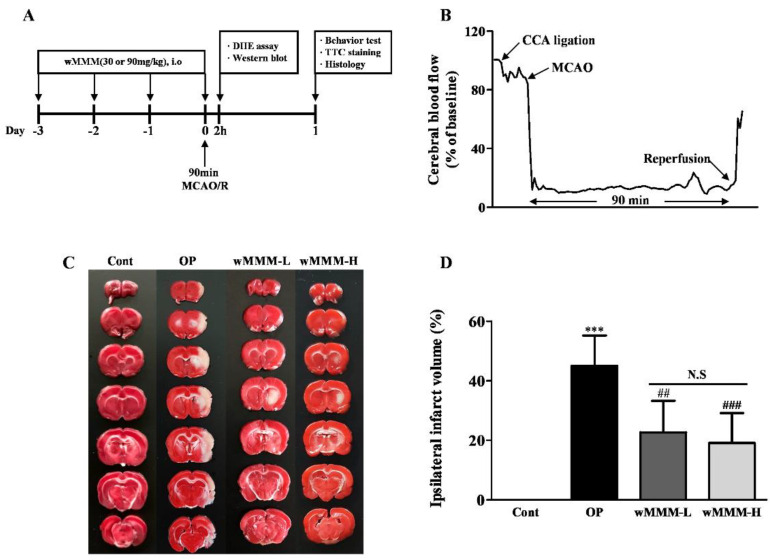
Reduction in infarct volume in in vivo focal cerebral ischemia (fCI) rat models by water-mixed mushroom mycelia (wMMM) administration. Time flow of the in vivo experimental schedule (**A**) and representative doppler flowmetry (**B**) showing changes in relative cerebral blood flow during the entire MCAO/R procedure. The details are presented in the Materials and Methods section. Representative photographs of 2,3,5-triphenyltetrazolium chloride-stained coronal brain sections (**C**) and the bar graph (**D**) quantifying ipsilateral infarct volume. In (**C**), the viable area is in red, whereas the infarcted area is in white (*** *p* < 0.001 vs. Cont; ^##^ *p* < 0.01 and ^###^ *p* < 0.001 vs. OP; N.S; not significant). Data are presented as the mean ± standard deviation. Significance was determined by one-way ANOVA followed by a Tukey post hoc test for pairwise comparisons (n per group = 6). DHE, dihydroethidium; MCAO/R, middle cerebral artery occlusion, and reperfusion; CCA, common cerebral artery; Cont, control group; OP, group with vehicle and MCAO/R; wMMM-L, the group with low dose (30 mg/kg) of wMMM and MCAO/R; wMMM-H, the group with high dose (90 mg/kg) of wMMM and MCAO/R.

**Figure 5:**
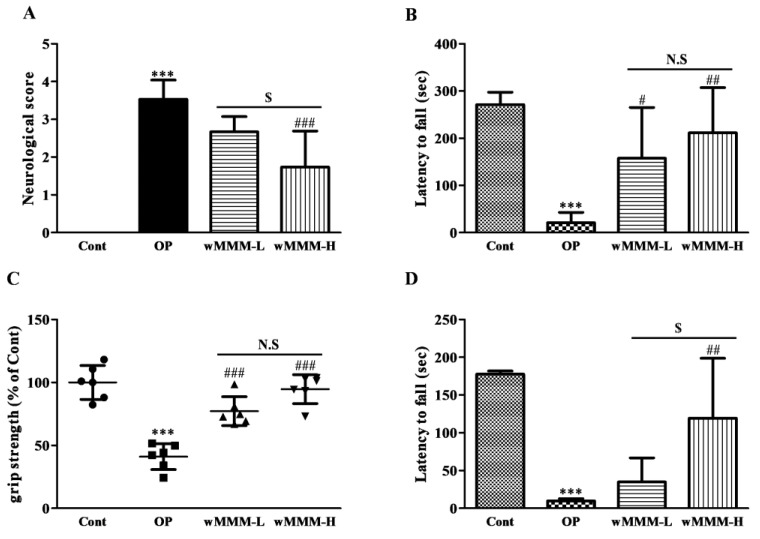
Attenuation of focal cerebral ischemia-induced sensorimotor deficits by water extracts of mixed mushroom mycelia (wMMM) administration. The graphs quantifying results from the neurological deficit scoring (**A**), the Rotarod test (**B**), the grip strength test (**C**), and the inverted screen test (**D**) were presented. In all graphs, data were presented as the mean ± standard deviation (*** *p* < 0.001 vs. Cont; ^#^ *p* < 0.05, ^##^ *p* < 0.01, and ^###^ *p* < 0.001 vs. OP; ^$^ *p* < 0.05 vs. wMMM-L; N.S, not significant). In all graphs, significance was determined by one-way ANOVA followed by a Tukey post hoc test for pairwise comparisons (n per group = 6). Cont, control group; OP, the group with vehicle and middle cerebral artery occlusion and reperfusion (MCAO/R); wMMM-L, the group with low dose (30 mg/kg) of wMMM; and MCAO/R; wMMM-H, the group with high dose (90 mg/kg) of wMMM and MCAO/R.

Next, we tested whether the wMMM could minimize the fCI-induced infarct volume and sensorimotor deficits using rats subjected to a MCAO/R to generate an in vivo fCI model. The wMMM at a dose of 30 or 90 mg/kg was orally administered four times prior to the operation, yielding the wMMM-L and wMMM-H group, respectively, and the resulting infarct volumes were measured at 24 h after the operation (Figure 4A). During the operation, the rCBF values of each individual were confirmed to have dropped below 20% of baseline by Doppler flowmetry. As shown in the representative flowmetry changes (Figure 4B), all groups had identical rCBF values during periods of 90 min of ischemia, indicating that brain perfusion was not affected by wMMM administration (data not shown). As shown in TTC-stained tissues (Figure 4C), while there were no visible areas of infarction in the Cont group, as expected, the OP group exhibited marked areas of infarction at 24 h after MCAO/R (*** *p* < 0.001). The ipsilateral infarct volumes in both the wMMM-L and wMMM-H groups were around half of the OP group (22.68 ± 9.46 and 19.11 ± 8.94 vs. 44.97 ± 9.44%, ^##^ *p* < 0.01 and ^###^ *p* < 0.001, respectively; Figure 4D). In addition, the extent of their reduction was not statistically different between these groups. Four different behavioral tests were conducted to assess the effects of wMMM on fCI-associated sensorimotor deficits. First, NDS demonstrated that the OP group displayed apparent sensorimotor deficits (3.53 ± 0.45, *** *p* < 0.001 vs. Cont; Figure 5A), which were significantly decreased in the wMMM-H group (1.73 ± 0.85, ^###^ *p* < 0.001 vs. OP group). Second, the Rotarod test revealed that the OP group showed a marked impairment in motor coordination (20.71 ± 20.36 vs. 271.20 ± 23.54, *** *p* < 0.001 vs. Cont; Figure 5B), which was significantly spared in the wMMM-L and wMMM-H groups (157.40 ± 96.34 and 211.40 ± 85.52, ^#^ *p* < 0.05 and ^##^ *p* < 0.01 vs. OP, respectively). The other two tests, the grip strength test (Figure 5C) and the inverted screen test (Figure 5D), indicated that the OP group showed weakened muscle strength (*** *p* < 0.001 vs. Cont), which was significantly spared by wMMM pretreatment (^##^ *p* < 0.01 and ^###^ *p* < 0.001 vs. OP). Notably, the results from NDS and the inverted screen test demonstrated that the wMMM-induced effects on muscle strength preservation were dose-dependent (^$^ *p* < 0.05). These findings demonstrated that wMMM could reduce the infarct volume and improve the sensorimotor deficits seen in fCI rats.

### 3.4. Water Extracts of Mixed Mushroom Mycelia (wMMM) Attenuates Cell Death and Apoptosis in an Focal Cerebral Ischemia (fCI) Lesion

**Figure 6:**
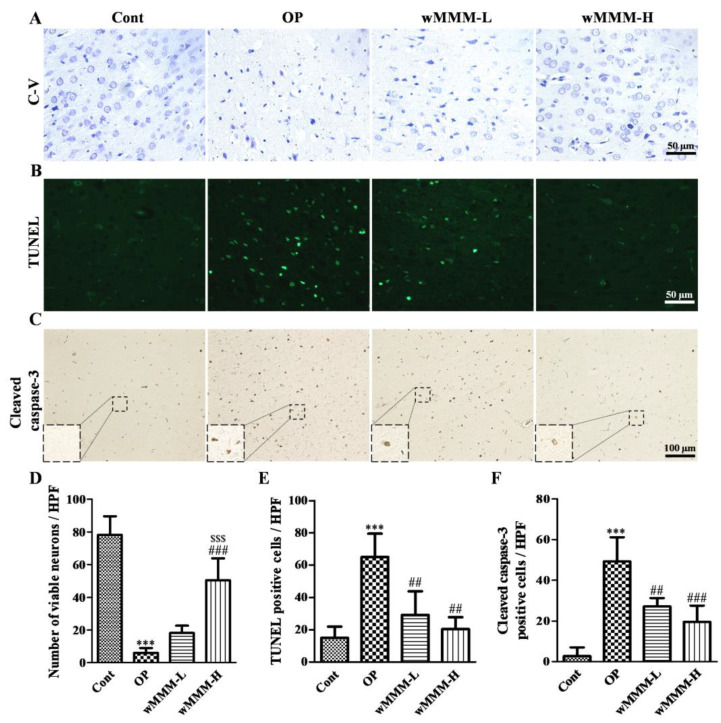
Anti-apoptotic effects of water extracts of mixed mushroom mycelia (wMMM) on an in vivo focal cerebral ischemia (fCI) model. Representative C-V stained (**A**), TUNEL-stained (**B**), and Cleaved caspase-3-immunostained (**C**) images of an fCI cortical lesion and the corresponding quantitative bar graphs (**D**–**F**). For viable neuronal counts shown in (**D**), only neurons with an intact cytoplasm and visible nucleolus were considered “viable”. In (**C**), the cleaved caspase-3-immunoreactive cells have been magnified and presented in rectangular inlets as indicated. In all bar graphs, data are presented as the mean ± standard deviation (*** *p* < 0.001 vs. Cont; ^##^ *p* < 0.01 and ^###^ *p* < 0.001 vs. OP; ^$$$^ *p* < 0.001 vs. wMMM-L). Significance was determined by one-way ANOVA followed by a Tukey post hoc test for pairwise comparisons (n per group = 6). C-V, cresyl-violet; TUNEL, terminal deoxynucleotidyl transferase dUTP nick-end labeling; Cont, control group; OP, the group with vehicle and MCAO/R; wMMM-L, the group with low dose (30 mg/kg) of wMMM and middle cerebral artery occlusion and reperfusion (MCAO/R); wMMM-H, the group with high dose (90 mg/kg) of wMMM and MCAO/R.

The C-V stain was employed to assess the extent of neuronal death at 24 h after MCAO/R. When compared with intact cortical tissue of the Cont, the OP group demonstrated the apparent loss of viable neurons with an intact cytoplasm and visible nucleolus in fCI cortical lesion (Figure 6A,D, *** *p* < 0.001). Although the wMMM-L group failed to inhibit neuronal loss in an fCI lesion, the wMMM-H group showed significant preservation of viable neurons (50.4 ± 11.96 vs. 6.00 ± 2.61, ^###^ *p* < 0.001 vs. OP). Given that apoptosis is known to be a primary mode of death in the context of an fCI lesion [3], TUNEL staining and immunohistochemical detection of Cleaved caspase-3, a key protein executioner responsible for apoptosis [48], were employed to assess the role of wMMM in apoptosis. As shown in Figure 6B,E, TUNEL-positive apoptotic cells were scarce in the intact brain cortical tissue of the Cont group, as expected. However, the OP group displayed a marked increase in TUNEL-positive cells in an fCI cortical lesion (*** *p* < 0.001 vs. Cont), which were significantly reduced in both groups pretreated with wMMM (29.25 ± 12.74 and 20.5 ± 6.34 in wMMM-L and -H group, respectively, vs. 65.00 ± 12.55; ^##^ *p* < 0.01 vs. OP). Additionally, as shown in Figure 6C, F, increases in the number of Cleaved caspase-3-immunoreactive cells (magnified in rectangular inlets, Figure 6C) in the fCI cortical lesion were clearly found, as expected (*** *p* < 0.001 vs. Cont). However, when compared with the OP group, the number of Cleaved caspase-3-immunoreactive cells in an fCI cortical lesion was markedly decreased in the wMMM-L and wMMM-H groups (27.20 ± 3.66 and 19.60 ± 7.12, respectively, vs. 49.20 ± 10.68, ^##^ *p* < 0.01 and ^###^ *p* < 0.001 vs. OP). These data suggest that wMMM-induced brain protection against fCI injury involves apoptosis attenuation.

### 3.5. Water Extracts of Mixed Mushroom Mycelia (wMMM) Attenuates Focal Cerebral Ischemia (fCI)-Associated Oxidative Stress and Upregulates Antioxidant Enzyme Levels

**Figure 7:**
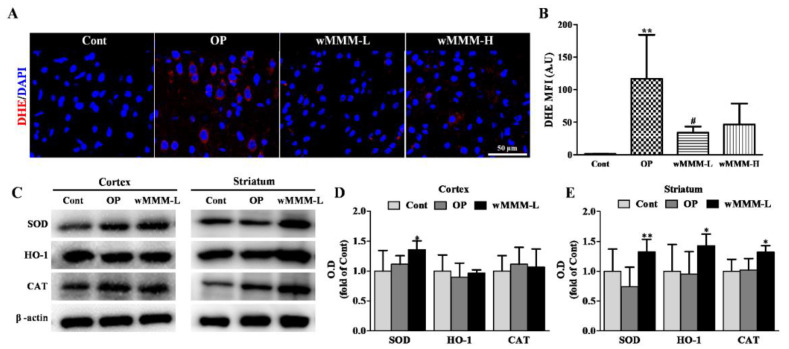
Antioxidant effects of water extracts of mixed mushroom mycelia (wMMM) on an in vivo focal cerebral ischemia (fCI) model. Representative images (**A**) of a DHE-stained fCI cortical lesion and the quantitative graph (**B**) showing the mean fluorescence intensity of DHE were presented. In (**A**), 4′,6-diamidino-2-phenylindole (DAPI) was used to stain nuclei. In (**B**), data are presented as the mean ± standard deviation (** *p* < 0.01 vs. Cont; ^#^ *p* < 0.05 vs. OP; n per group = 2). (**C**) Representative Western blot images showing the amount of SOD, HO-1, and CAT in the ischemic cortex or striatum homogenates and the corresponding bar graphs (**D**,**E**, respectively). For the quantifications in (**D**,**E**), the optical density was normalized by β-actin. In (**D**,**E**), data are presented as the mean ± standard deviation (* *p* < 0.05 and ** *p* < 0.01 vs. OP; n per group = 4). In all graphs, significance was determined by one-way ANOVA followed by a Tukey post hoc test for pairwise comparisons. DHE, dihydroethidium; A.U, arbitrary unit; SOD, Superoxide dismutase; HO-1, heme oxygenase-1; CAT, Catalase; Cont, control group; OP, the group with vehicle and middle cerebral artery occlusion and reperfusion (MCAO/R); wMMM-L, the group with low dose (30 mg/kg) of wMMM and MCAO/R; wMMM-H, the group with high dose (90 mg/kg) of wMMM and MCAO/R.

Having established that wMMM can reduce fCI-associated brain cellular apoptosis, we performed further studies to assess the involvement of wMMM-mediated ROS attenuation, as shown in vitro (Figure 3E,F), in the therapeutic mechanisms. To this end, we employed in vivo staining with DHE, a well-known fluorescence marker for superoxide [49]. The resulting red fluorescence was quantitatively assessed at 2 h after MCAO/R in different groups. As shown in Figure 7A,B, cellular ROS accumulation (red) in an fCI cortical lesion was markedly increased by 2 h after MCAO/R in the OP group (116.59 ± 58.55, *p* ** < 0.01 vs. Cont). Although there was no significant difference between the wMMM-H and OP groups, the wMMM-L group exhibited a marked reduction in DHE-fluorescence when compared with the OP group (33.97 ± 8.20, *p* ^#^ < 0.05). Considering the crucial role of endogenous antioxidant enzyme levels on the clearance of intracellular ROS and that the upregulation of the enzymes is commonly associated with antioxidant activity exerted by naturally-originated compounds, we next quantified the levels of SOD, HO-1, and CAT, which are well-known key antioxidant enzymes in brain cells [50,51]. As we observed that wMMM exerted the antiapoptitic activity against fCI, even at low levels (30 mg/kg), cortical and striatal homogenates from the Cont, OP, and wMMM-L groups were differentially prepared and their enzyme contents were comparatively analyzed using Western blot techniques. As shown in the representative band images (Figure 7C) and the quantification graphs (Figure 7D,E), levels of SOD, HO-1, and CAT in both regions remained unchanged in the OP groups compared with the Cont group. Notably, although HO-1 and CAT failed to be upregulated in cortices, cortical SOD and striatal SOD, HO-1, and CAT were significantly increased in the wMMM-L group compared with the OP group (1.36 ± 0.14 vs. 1.12 ± 0.13, 1.33 ± 0.19 vs. 0.74 ± 0.29, 1.43 ± 0.17 vs. 0.95 ± 0.33, and 1.32 ± 0.10 vs. 1.02 ± 0.17, respectively, * *p* < 0.05 and ** *p* < 0.01). Together, these results suggest that wMMM intake can attenuate the fCI-induced oxidative damage and, at least in part, the wMMM-mediated upregulation of antioxidant enzymes might contribute to its anti-oxidative properties.

## 4. Discussion

Stroke, mainly in the form of fCI, is the second most common cause of death and the leading cause of adult neurological disability worldwide [2]. Associated events include the release of excitatory amino acids such as glutamate [Ca^2+^]_i_, overload, energy depletion, ROS accumulation, and neuro-inflammation; all of which culminate in apoptotic neuronal death [52,53]. Among these, ROS-triggered oxidative damage and neuro-inflammation are thought to be key detrimental contributors to fCI-induced neuronal apoptosis [54]. With regard to the role of ROS in apoptosis, over-produced ROS can cause protein modification or degradation through interactions with amino acids in protein molecules. In addition, it can induce DNA mutations or structural changes by breaking DNA double strands [55]. As for neuro-inflammation, microglia, and astrocytes that become activated during the process of fCI then participates in subsequent inflammation by secreting pro-inflammatory cytokines, Interleukin-1β (IL-1β), IL-6, and Tumor necrosis factor-α (TNF-α), i.e., key cytokines are produced by these cells in response to fCI pathology [53]. The resulting apoptosis, which is seen in an infarct’s core immediately after fCI, lasts for several days, resulting in a gradual expansion of the infarct area from the infarct core to the ischemic penumbra [56]. Given the above, strategies to attenuate oxidative stress in neurons and inflammatory response in the glia, including microglia and astrocytes, are believed to be helpful in the treatment or prevention of fCI.

A large body of evidence indicates that edible mushrooms possess various health-promoting properties including anticancer, antibacterial, antifungal, antiviral, immunomodulating, antiallergic, antidepressive, antihyperlipidemic, antidiabetic, hepatoprotective, nephroprotective, osteoprotective, and hypotensive activities in addition to, remarkably, anti-oxidative activities [17,18,19,20,21,22,23,24,57]. Among mushrooms, PL, GL, and IO are reported to have outstanding neuroprotective effects [32,33,34]. PL extract has dose-dependent protective effects on oxidative stress-induced apoptosis by diminishing Cleaved caspase-3 and ROS levels and increasing the expression of HO-1, CAT, and SOD in SK-N-MC cells, a human neuroblastoma cell line [58]. In the case of GL, it has been reported to protect cultured cerebellar granule cells against H_2_O_2_-a ROS donor-induced apoptosis by decreasing the expression of caspase-3, Bax, and Bim and increasing that of Bcl-2 [59]. As for IO, its major triterpenoid constituents have been demonstrated to have protective efficacy in SH-SY5Y cells, a neuron-like neuroblastoma cell line, against H_2_O_2_-induced neurotoxicity [60].

Together, these results demonstrate the strong possibility of a synergistic augmentation of neuroprotective effects when PL, GL, and IO are supplied in combination. However, these experimental trials have been scarce due to their rareness in nature, difficulties with the methods, and the resulting expenses to individuals. However, owing to modern SSF-based cultivation techniques, the production of mixed mushrooms such as the PL, GL, and IO as a form of mycelia and the experiments carried out to screen for possible neuroprotection have become possible. Using the unique cultivation and preparation methods supplied by our colleague (Giunchan, Co., Ltd., Cheonan-si, Korea), we demonstrated that the consumption of w-MMM, e-MMM, and ea-MMM has neuroprotective effects on an in vitro fCI model, which was established with glutamate-induced excitotoxic cell death using PC-12 cells. Furthermore, among these, our findings revealed that wMMM was the safest and the most efficacious fraction.

In order to identify the compounds that are prevalent in the wMMM, a chemical profiling using an LC-MS was performed. Although additional chemistry-based analyses are needed, the resulting nine chemical constituents were suspected to be Sanleng acid, 9-Ene-methyl palmitate, Dibutyl sebacate, Matairesinoside, 19-Glucosyl-14-deoxyandrographolide, Vernolic acid, 6-Gingerol, n-Henicosanal, and Kingianoside B presented in increasing order of their retention times (Table 1). Information on the biological effects of the individual compounds remain largely limited; however, the effects of 6-Gingerol are relatively well-studied. 6-Gingerols, which is a phenolic ingredient first purified from ginger, has been reported to exert anti-inflammatory [61] and anti-oxidative effects [62]. Remarkably, in the era of neuroprotection, 6-Gingerol has been demonstrated to protect against hypoxia-induced apoptosis of PC-12 cells by upregulating the neuroprotective microRNA (miR) miR-103 [63]. Furthermore, recent evidence has shown that 6-Gingerol can attenuate lipopolysaccharide-induced neuro-inflammation in vitro and in vivo by suppressing astrocyte overactivation [61]. Recent studies have suggested that therapeutic mechanisms underlying wMMM-induced neuroprotection against fCI might involve, at least in part, 6-Gingerol-mediated anti-apoptotic-inflammatory and anti-neuro-inflammatory properties. Considering that the strategic reinforcement of the target compound ingredients is possible using the SSF-based MMM cultivation method, various modifications to culture conditions (e.g., mixing ratio of the three MMM, temperature, and duration) aimed at 6-Gingerol augmentation might represent a viable strategy.

Our study has some limitations. Since our study only focused on identifying the roles of the therapeutic effect of wMMM alone without comparing that of PL, GL, and IO on fCI injury in vitro and in vivo, we were unable to quantify the actual synergism by the combination of three MMM. In order to confirm the superiority in terms of therapeutic effects of wMMM, compared with those of water extracts of PL, GL, and IO, more detailed comparative analyses using the above variants as an experimental control are essentially required. Furthermore, the possible upstream targets of the antioxidant enzyme upregulated by wMMM were not assessed. For example, the nuclear factor erythroid-derived 2-related factor 2 (Nrf2) is the most powerful candidate as an upstream target given that it is the master regulator of anti-oxidative enzymes, including SOD, HO-1, and CAT [51]. Under healthy conditions, a cytosolic Nrf2 is sequestered by binding to Kelch-like ECH-associated protein 1 (Keap1), which inhibits translocation to the nucleus [64]. Several forms of stimuli, such as hypoxia, trigger the conformational change of Keap1 that enables the Nrf2/Keap1 complex to release Nrf2, which then translocates to the nucleus and binds to the promoter regions of antioxidant genes [65,66]. This leads us to assume that the activation of the Nrf2/Keap1 pathway is involved in the wMMM-mediated neuroprotective effects against fCI. As such, a more detailed analysis of the possible modulation of wMMM on Nrf2/Keap1 will be important in further investigations.

In conclusion, our findings revealed that wMMM can attenuate apoptotic cell death against glutamate-induced excitotoxicity in vitro and minimize the infarct volume and sensorimotor deficits followed by fCI in vivo by attenuating ROS accumulation and upregulating antioxidant enzyme levels. As such, one of the underlying mechanisms includes the exertion of anti-oxidative effects. In this regard, we strongly suggest that a wMMM supplement may be a useful preventive strategy against fCI.

## Figures and Tables

**Table 1:** Mass spectrometric data (retention time, molecular formula, molecular weight, and charge-to-mass ratio) of the verified compounds.

Serial Number	Retention Time (Min)	Molecular Formula	Molecular Weight (Da)	[M-H]^−^ (*m*/*z*)	Compound
1	7.97	C_18_H_34_O_5_	330.2406	329.2329	Sanleng acid
2	9.36	C_17_H_32_O_2_	268.2402	313.2379	9-Ene-methyl palmitate
3	9.84	C_18_H_34_O_4_	314.2457	313.2371	Dibutyl sebacate
4	10.34	C_26_H_32_O_11_	520.1945	565.1916	Matairesinoside
5	10.70	C_26_H_40_O_10_	512.5963	541.268	19-Glucosyl-14-deoxyandrographolide
6	11.14	C_18_H_32_O_3_	296.2351	295.2272	Vernolic acid
7	13.69	C_17_H_26_O_4_	294.1831	293.1763	6-Gingerol
8	14.27	C_21_H_44_	310.3236	355.3208	n-Henicosanal
9	14.47	C_30_H_60_O_13_	736.4034	735.3934	Kingianoside B

## Data Availability

Not applicable.

## Supplementary Materials

The following are available online at https://www.mdpi.com/article/10.3390/cimb43010030/s1.
