# Supplementary material for: Water Extract of Mixed Mushroom Mycelia Grown on a Solid Barley Medium Is Protective against Experimental Focal Cerebral Ischemia
Source: Curr Issues Mol Biol. 2021 Jun 15;43(1):365–83. doi: 10.3390/cimb43010030 (PMC8928960; doi:10.3390/cimb43010030)
Supplement: Supplementary file 1 [file cimb-43-00030-s001.zip › cimb-1235026-supplementary.pdf]

Supplementary Table S1. List of the antibodies and other reagents.

Antibodies			
**Product**	**application**	**Supplier**	**Catalogue #**
Rabbit Cleaved caspase-3 (Asp175) antibody	IHC	Cell signaling	9661s
Rabbit (DA1E) mAB IgG XP Isotype Control	IHC	Cell signaling	3900s
Goat anti-rabbit IgG, biotinylated	IHC	Vector laboratories	BA-1000-1.5
Rabbit Superoxide dismutase (SOD) antibody	WB	Sigma-Aldrich	S5569
Rabbit Heme oxygenase-1 (HO-1) antibody	WB	Abcam	Ab68477
Rabbit Catalase (CAT) antibody	WB	Sigma-Aldrich	219010
Mouse β-actin antibody	WB	Santa Cruz	SC-47778
Goat anti-Mouse IgG, HRP	WB	Invitrogen	31430
Goat anti-Rabbit IgG, HRP	WB	Invitrogen	31460

Other Reagents			
**Product**	**application**	**Supplier**	**Catalogue #**
Pierce™ BCA Protein Assay Kit	WB	Thermo-Fisher	23227
Hoechst33258	Histofluorescence	Sigma-Aldrich	861405
2', 7'-dichlorodihydrofluorescin diacetate (DCF-DA)	ROS (*in vitro*)	Sigma-Aldrich	D6883
3-(4,5-dimethylthiazol-2-yl)-2,5-diphenyl tetrazolium bromide (MTT)	Cell viability	Sigma-Aldrich	M2128
Annexin V-FITC Apoptosis Detection Kit	Apoptosis	Merck-Millipore	CBA059
TUNEL assay Kit	Apoptosis	Promega	G3250
Triphenyltetrazolium chloride (TTC)	Infarction size	Sigma-Aldrich	T8877
Cresyl-violet acetate (C-V)	Neuron count	Sigma-Aldrich	C5042
VECTASTAIN® ABC Kits	IHC	Vector laboratories	PK6100
3,3'-Diaminobenzidine (DAB)	IHC	Vector laboratories	SK4100
Dihydroethidium (DHE)	ROS (*in vivo*)	Invitrogen	D1168
